# The Role of Artificial Intelligence in Early Diagnosis and Molecular Classification of Head and Neck Skin Cancers: A Multidisciplinary Approach

**DOI:** 10.3390/diagnostics14141477

**Published:** 2024-07-10

**Authors:** Zeliha Merve Semerci, Havva Serap Toru, Esra Çobankent Aytekin, Hümeyra Tercanlı, Diana Maria Chiorean, Yalçın Albayrak, Ovidiu Simion Cotoi

**Affiliations:** 1 Department of Oral and Maxillofacial Radiology, Faculty of Dentistry, Akdeniz University, 07070 Antalya, Turkey; merveertugrul@outlook.com (Z.M.S.); ysl_hmyr25@hotmail.com (H.T.); 2 Department of Pathology, Faculty of Medicine, Akdeniz University, 07070 Antalya, Turkey; 3 Department of Pathology, Konya Numune Hospital, 42060 Konya, Turkey; esracobankent@hotmail.com; 4 Department of Pathology, County Clinical Hospital of Targu Mures, 540072 Targu Mures, Romania; chioreandianamaria@yahoo.com (D.M.C.); ovidiu.cotoi@umfst.ro (O.S.C.); 5 Department of Pathophysiology, “George Emil Palade” University of Medicine, Pharmacy, Science, and Technology of Targu Mures, 38 Gheorghe Marinescu Street, 540142 Targu Mures, Romania; 6 Department of Electric and Electronic Engineering, Faculty of Engineering, Akdeniz University, 07010 Antalya, Turkey; yalbayrak32@gmail.com

**Keywords:** head and neck neoplasms, artificial intelligence, skin neoplasms, diagnostic imaging, diagnostic molecular pathology

## Abstract

Cancer remains a significant global health concern, with increasing genetic and metabolic irregularities linked to its onset. Among various forms of cancer, skin cancer, including squamous cell carcinoma, basal cell carcinoma, and melanoma, is on the rise worldwide, often triggered by ultraviolet (UV) radiation. The propensity of skin cancer to metastasize highlights the importance of early detection for successful treatment. This narrative review explores the evolving role of artificial intelligence (AI) in diagnosing head and neck skin cancers from both radiological and pathological perspectives. In the past two decades, AI has made remarkable progress in skin cancer research, driven by advances in computational capabilities, digitalization of medical images, and radiomics data. AI has shown significant promise in image-based diagnosis across various medical domains. In dermatology, AI has played a pivotal role in refining diagnostic and treatment strategies, including genomic risk assessment. This technology offers substantial potential to aid primary clinicians in improving patient outcomes. Studies have demonstrated AI’s effectiveness in identifying skin lesions, categorizing them, and assessing their malignancy, contributing to earlier interventions and better prognosis. The rising incidence and mortality rates of skin cancer, coupled with the high cost of treatment, emphasize the need for early diagnosis. Further research and integration of AI into clinical practice are warranted to maximize its benefits in skin cancer diagnosis and treatment.

## 1. Introduction

Skin cancer is globally recognized as the most prevalent form of cancer and comprises about one-third of all cancers diagnosed [1]. Despite an upward trend in incidence rates over recent years, mortality rates have not significantly changed [2]. Skin cancers are primarily categorized into malignant melanoma (MM) and non-melanoma skin cancer (NMSC). Women and people aged 61 to 90 years face a higher risk of NMSC, and the majority of skin cancers of the head and neck region are NMSCs [3]. NMSC itself includes two principal subtypes: basal cell carcinoma (BCC) and squamous cell carcinoma (SCC) [4,5,6]. BCC originates from the basal layer’s epidermal cells and represents around 80% of skin cancer cases, making it the most common type. The ratio of BCC to SCC is 3 to 4:1 [7]. Although BCC grows slowly and is typically non-aggressive, leading to rare metastasis, it can still cause considerable morbidity and complications due to its capacity for local invasion [8,9]. SCC, accounting for approximately 20% of all NMSC cases, emerges from the malignant proliferation of epidermal keratinocytes and exhibits a more aggressive behavior with an increased risk of metastasis [9,10,11,12]. The primary etiological factor for non-melanoma skin cancer is extensive exposure to ultraviolet radiation (UVR), particularly within the 290 to 320 nm spectrum, noted for its potent carcinogenic effects [7,13]. Additional risk factors include exposure to radiation, long-term arsenic ingestion, and susceptibility in individuals with fair skin who are prone to sunburns. NMSC can also develop from pre-existing conditions such as scars and chronic ulcers [14]. Diagnosing skin cancer can be particularly challenging due to the differential diagnosis that must distinguish it from other dermatological conditions, such as benign nevi, seborrheic keratosis, and actinic keratosis. These conditions can resemble skin cancer in their clinical presentation, making accurate diagnosis difficult. The propensity of skin cancer to metastasize highlights the importance of early detection for successful treatment [15]. Furthermore, individuals with genetic disorders such as xeroderma pigmentosum, an autosomal-recessive disorder characterized by diminished DNA repair capabilities, and nevoid basal cell carcinoma syndrome, an autosomal-dominant condition associated with multiple basal cell carcinomas and other physical anomalies, are at an increased risk [16]. Management of NMSC involves early detection, ongoing UV protection, and continuous skin surveillance. Notably, there is a higher occurrence and aggressive nature of squamous cell carcinoma in the head and neck region among organ transplant recipients, with a significant propensity for metastasis [17].

In contrast, MM, which develops from melanocytes, the skin’s pigment-producing cells, is the least common but most severe form of skin cancer, contributing to the highest number of skin cancer-related deaths. MM accounts for only 1% of skin cancer cases but poses significant mortality risks. According to the American Cancer Society, MM ranks as the sixth most common cancer in the United States. The primary risk factor for NMSC is prolonged exposure to ultraviolet radiation, predominantly affecting skin areas frequently exposed to sunlight, such as the head and neck [18,19,20]. The risk of developing melanoma is notably higher in individuals who experienced severe sunburns during their early years, particularly those with fair skin, blue eyes, red or blond hair, light complexions, and a tendency to freckle. It is critical to initiate sun protection education early for these at-risk populations. Most malignant melanomas originate in normal skin, not from existing melanocytic lesions. Research indicates that 4% to 72% of melanomas are histologically linked to a large count of melanocytic nevi, including common and dysplastic types, with the highest frequency between 20% and 30% [7,21]. Congenital melanocytic nevi (CMN) present at birth can also develop into melanoma, with a particularly high risk noted in giant CMN. Lentigo maligna, considered a melanoma precursor, has a variable progression rate to lentigo maligna melanoma, with lifetime risks calculated at different age milestones [22,23]. Familial history, especially coupled with dysplastic nevi, significantly elevates melanoma risk, underscoring the necessity for thorough initial assessments, consistent monitoring, and selective biopsies to diagnose dysplastic conditions and potential melanomas. Additionally, xeroderma pigmentosum remains a genetic condition closely linked to melanoma development [16].

Skin cancer represents one of the most aggressive cancer types, with a significant propensity for metastasis to lymph nodes and other organs. For effective clinical management and treatment, it is crucial to determine the extent and specific sites of metastatic spread accurately. This process, termed ‘disease staging,’ is essential for identifying whether skin cancer has involved regional lymph nodes or disseminated to distant organs, such as the liver or brain. Advanced imaging modalities are indispensable for assessing the extent of metastasis. Recent therapeutic developments offer promising strategies to mitigate the risk of metastasis and manage advanced stages of skin cancer [24].

The diagnosis of skin cancer involves several methodologies, each with unique benefits and limitations regarding diagnostic accuracy and practical implementation (Table 1). Visual examination, where dermatologists assess the skin for suspicious moles or lesions, serves as a primary, non-invasive, rapid, and accessible screening tool. Biopsy, the gold standard for diagnosis, entails the excision of a skin tissue sample for histopathological analysis [25]. Dermoscopy enhances diagnostic accuracy by providing more detail than visual examination and is also non-invasive, although it requires training and its interpretation can still be subjective. For instance, BCC often shows arborizing telangiectasia, leaf-like areas, and ovoid nests; SCC typically presents with white structureless areas, dotted or glomerular vessels, and scale; melanoma is identified by asymmetry, irregular borders, multiple colors, and structures like an atypical network, blue-white veil, and irregular streaks [26]. Molecular pathology involves the analysis of genetic and molecular markers within the skin tissue. This technique provides vital information on genetic mutations and prognosis, facilitating precise cancer characterization. Although highly informative, molecular pathology necessitates specialized equipment and is costly, limiting its widespread application. It is particularly valuable for identifying mutations that inform targeted therapies. The diagnostic accuracy of molecular techniques is high, especially in determining genetic profiles, but they are often used adjunctively with other diagnostic methods [27,28].

High-frequency ultrasound (HFUS) utilizes high-frequency sound waves to generate detailed images of skin structures. It is particularly effective for evaluating the depth and extent of skin tumors, which is critical for staging and treatment planning. HFUS provides a high resolution, typically between 20 and 100 MHz, but it cannot visualize cellular details, limiting its ability to distinguish between benign and malignant lesions. For melanoma, ultrasound is instrumental in measuring tumor thickness (Breslow thickness), which is essential for cancer staging and determining surgical margins [29,30]. Additionally, it is used to evaluate regional lymph nodes for metastasis, with suspicious nodes being further examined via a fine-needle aspiration biopsy under ultrasound guidance. The non-invasive nature of ultrasound and its ability to provide detailed, real-time images of the skin and underlying tissues makes it an invaluable tool. Measuring Breslow thickness, the tumor’s depth from the top layer of the epidermis to its deepest point, is crucial for staging melanoma and planning treatment [31]. Ultrasound can also detect dermal infiltration, indicating the spread of cancer cells into the dermal layer, which is significant for staging and determining aggressiveness. Irregular margins on ultrasound suggest invasive tumors, common in aggressive melanomas and squamous cell carcinomas. Ultrasound also assesses regional lymph nodes for metastasis, with enlarged, hypoechoic lymph nodes potentially indicating metastatic involvement, which is crucial for staging melanoma and SCC [32]. Basal cell carcinoma lesions typically present as well-defined oval or slightly irregular hypoechoic structures, often containing hyperechoic spots. Doppler ultrasound frequently detects low-flow arterial and venous vessels within or at the base of these lesions, with the presence of tortuous vessels suggesting the possibility of different tumor types. Elastography generally indicates that BCCs are relatively stiff [33,34]. Conversely, squamous cell carcinoma is identified on ultrasound as a heterogeneously hypoechoic lesion with irregular borders, devoid of hyperechoic spots, and tends to invade deeper tissue layers. SCCs exhibit a diffusely increased low-flow vascular pattern, particularly accentuated at the lesion’s periphery [35]. Melanomas on ultrasound appear as well-defined oval or fusiform, homogeneous, echo-poor lesions. The hyperechoic epidermal line is typically observed above the tumor, except in ulcerated cutaneous melanomas, with increased acoustic transmission being a common feature [36]. The effectiveness of ultrasound depends on the availability of high-quality equipment and the expertise of the operator, with dermatologists and radiologists trained in dermatologic ultrasound providing the most accurate assessments. While ultrasound is valuable on its own, it is often used in combination with other imaging modalities such as dermoscopy, MRI, and CT scans for a comprehensive assessment and staging [32].

Spectroscopy techniques, such as Raman and Fourier Transform Infrared (FTIR) spectroscopy, analyze the molecular composition of skin tissues by measuring light interactions with biological molecules. These techniques provide detailed biochemical information, allowing differentiation between cancerous and non-cancerous cells with high sensitivity and specificity, often exceeding 90%. However, they are primarily used in research due to their complexity and cost [31,37]. Computed Tomography (CT) scans use X-rays to produce detailed cross-sectional images, which are invaluable for staging skin cancer and detecting metastasis. CT can identify cancer spread to lymph nodes and organs with high accuracy, with sensitivity and specificity rates around 80–90%. While not typically used for primary diagnosis of skin lesions, it plays a crucial role in comprehensive cancer staging. Magnetic Resonance Imaging (MRI) utilizes strong magnetic fields and radio waves to create detailed images of soft tissues. MRI is especially useful for assessing tumor depth and detecting metastasis in soft tissues and the central nervous system. It offers superior contrast resolution compared to CT, though it is more expensive. MRI’s diagnostic accuracy for staging and detecting metastasis is high, with sensitivity and specificity often exceeding 90% [31,38]. Optical Coherence Tomography (OCT) uses light waves to generate high-resolution cross-sectional images of skin tissues. Currently, OCT demonstrates the potential to detect numerous diseases. Research indicates that OCT enhances diagnostic accuracy in gastrointestinal disorders, cervical neoplasia, and oral cancers. OCT is effective for examining the epidermis and superficial dermis, making it useful for early melanoma detection. However, its limited penetration depth restricts its use to superficial lesions [37,39]. Optical techniques, such as dermoscopy, enhance the visual assessment of skin lesions through magnification and polarized light. These techniques improve diagnostic accuracy significantly when performed by trained clinicians, with sensitivity around 70–90% and specificity around 80–90%. The effectiveness of dermoscopy depends heavily on the clinician’s expertise and experience [26,40]. Photodynamic-based techniques involve using photosensitizing agents that accumulate in cancer cells. When exposed to specific wavelengths of light, these agents produce reactive oxygen species that selectively destroy malignant cells. Thermal imaging detects infrared radiation emitted by the skin to identify temperature variations associated with abnormal metabolic activity in cancerous tissues. This non-invasive method can indicate tumor presence, but it is less specific and can be influenced by external factors [41]. Reflectance confocal microscopy (RCM) generates high-resolution images of the skin’s epidermis and upper dermis, allowing non-invasive, in vivo examination. RCM improves the visualization of skin structures at a cellular level, enhancing the detection of malignant features without the need for tissue excision. Its non-invasive nature and repeatability make it an attractive option for monitoring suspicious lesions [42]. Digital pathology involves digitizing pathology slides for analysis and sharing, facilitated by artificial intelligence. This method enables remote consultations and enhances collaboration among healthcare providers. Digital pathology requires substantial digitization and storage infrastructure, making it resource-intensive. Its diagnostic accuracy is augmented by AI algorithms that can precisely analyze patterns and anomalies, reducing human error [43]. Integrating AI into digital pathology can enhance diagnostic consistency and efficiency, particularly in large-scale screening programs. AI-based image analysis employs advanced machine learning algorithms, such as convolutional neural networks (CNNs), to interpret dermatological images. These systems can identify patterns and anomalies that might be overlooked by human observers, achieving high diagnostic accuracy with sensitivity and specificity rates often exceeding 90% [44]. AI’s ability to consistently analyze large datasets without fatigue makes it valuable for screening programs, though its effectiveness is contingent on the quality and diversity of training data. Radiomics extracts quantitative features from medical images to aid in predicting disease progression and treatment responses. By analyzing lesions’ texture, shape, and intensity, radiomics can enhance the predictive power of mostly cross-sectional imaging techniques. This approach has shown promise in improving accuracy for predicting lymph node metastasis in melanoma patients, but it remains in the early stages of clinical application and requires further validation for standardization [45,46].

Each diagnostic method provides unique advantages and varying degrees of accuracy, contributing to a comprehensive approach for diagnosing, monitoring, and treating skin cancer. Combining these techniques enhances diagnostic precision and treatment planning, ensuring optimal patient outcomes.

With the integration of artificial intelligence into the healthcare field, the landscape of diagnostic approaches for skin cancers has experienced substantial transformation. This integration has facilitated the development and refinement of advanced diagnostic tools, enhancing accuracy and efficiency in detecting and characterizing various skin malignancies. This study aims to address skin cancers from a diagnostic and pathological perspective and to discuss the role of artificial intelligence and deep learning technologies in diagnosing skin cancers alongside modern diagnostic approaches. The topic of skin cancer was chosen due to its high prevalence, particularly among the white population, and its significant treatment costs among other cancers [47,48,49].

## 2. Literature Search and Selection

In conducting this narrative review on the role of artificial intelligence in the early diagnosis and molecular classification of head and neck skin cancers, a comprehensive literature search was performed to identify relevant studies and articles. The search strategy focused on three primary databases: PubMed, Scopus, and Google Scholar. The keywords used in the search included “skin cancer”, “head and neck neoplasms”, “artificial intelligence”, “AI”, “early diagnosis”, “digital pathology”, and “molecular classification”. The initial search yielded many articles, which were then screened based on their titles and abstracts.

Inclusion criteria for the articles included:

- Publications that discussed the role of artificial intelligence in the diagnosis of skin cancers, specifically in the head and neck region.
- Studies that covered both radiological and pathological perspectives of skin cancer diagnosis.
- Articles published in English within the last two decades, given the rapid advancements in AI and its applications in medical imaging and diagnostics.

The exclusion criteria were as follows:

- Studies that focused solely on non-skin cancers.
- Articles that did not provide substantial information on the use of AI in diagnosis.
- Publications in languages other than English.

After the initial screening, full-text reviews of the selected articles were conducted to ensure their relevance and to extract detailed information about the methodologies, findings, and implications of AI applications in the diagnosis of head and neck skin cancers. The final selection included studies that highlighted significant advancements in AI technology, its integration into clinical practice, and its impact on improving diagnostic accuracy and patient outcomes.

## 3. Discussion

Artificial intelligence dramatically enhances our ability to mimic human perception, especially in image processing and object detection. AI advancements are critical for improving the effectiveness and efficiency of healthcare services. This narrative review explores the diagnostic and pathological aspects of skin cancers, highlighting the significant role of artificial intelligence and deep learning technologies in their diagnosis. It also discusses modern diagnostic approaches that complement these advanced technologies. Optimizing clinical operations is paramount with the growing need for medical services and the daily surge in data. AI excels in recognizing complex image patterns and converting subjective image analysis into a reproducible, measurable task. AI also integrates diverse data streams to create comprehensive diagnostic tools, combining radiographic images, genomic data, pathology results, electronic health records, and social network information [50,51]. AI-based imaging biomarkers have emerged as powerful tools in the diagnostic arsenal against skin cancer (Table 2).

These biomarkers, which include hand-crafted radiomic features and deep learning (DL)-derived features, provide quantitative measures that correlate with clinical outcomes. Radiomic features extracted from imaging data describe tumor characteristics such as shape, texture, and intensity, which can then be used in machine learning models to predict disease severity and treatment response. The success of these biomarkers in other cancer types suggests their applicability to skin cancer, where early and accurate diagnosis is crucial. Hand-crafted radiomic approaches involve the extraction of predefined quantitative features from medical images. These features, carefully selected by AI development teams comprising computer scientists, radiologists, and oncologists, capture critical aspects of tumor biology [44,52]. In skin cancer diagnostics, radiomics can enhance the detection and classification of lesions by analyzing their morphological and textural properties. For instance, radiomic features that describe the heterogeneity of skin lesions may provide insights into malignancy, thus aiding in early diagnosis and personalized treatment planning [45]. Deep learning models, particularly convolutional neural networks (CNNs), have demonstrated remarkable pattern recognition and image analysis proficiency. These models can be trained on large datasets to identify complex patterns in imaging data that may be indicative of skin cancer. For skin cancer, CNNs can be employed to analyze dermoscopic images, distinguishing between benign and malignant lesions with high accuracy [53].

One of the strengths of AI in oncology is its capacity to integrate imaging data with clinical and genomic information. Radiogenomics, which combines radiomic and genomic data, allows for the development of non-invasive imaging biomarkers that reflect the molecular profile of tumors. This integration is particularly valuable in skin cancer diagnostics, where genomic alterations often drive disease progression. By leveraging radiogenomic approaches, clinicians can obtain a comprehensive view of the tumor’s biological behavior, facilitating more precise and individualized treatment strategies [54,55].

Recent studies have reported that artificial intelligence enhances the diagnostic performance of clinicians in various cancers where early diagnosis is critical [56]. Several studies in the literature have used AI networks to diagnose skin cancer, yielding diverse results.

Ali and Al-Marzouqi [57] used a deep learning framework, LightNet, to classify benign and malignant skin lesions using the ISIC 2016 dataset. Their method, well suited for mobile applications due to fewer parameters, achieved an accuracy of 81.6%, a sensitivity of 14.9%, and a specificity of 98%. A study by Nasr-Esfahani et al. [58] employed a CNN classifier on 170 skin lesion images to differentiate between melanoma and benign lesions. They achieved an accuracy of 81%, a sensitivity of 81%, and a specificity of 80%. Dorj et al. [59] used an SVM with a deep CNN for classifying BCC, SCC, melanoma, and actinic keratosis (AK) on a dataset of 3753 dermoscopic images. They achieved accuracies of 95.1% for SCC, 98.9% for AK, and 94.17% for BCC. Esteva et al. [60] utilized a deep CNN on the ISIC Dermoscopic Archive to classify melanoma and benign lesions, keratinocyte carcinomas, and benign seborrheic keratosis. The system demonstrated expert-level performance with an accuracy of 72.1%. A study by Mendes et al. [61] used a CNN with Res-Net 152 architecture to classify malignant melanoma and BCC. Their datasets included 170 and 1300 images, respectively, achieving AUC values of 96% for melanoma and 91% for BCC.

Harangi et al. [62] employed a CNN ensemble of AlexNet, VGGNet, and GoogleNet to classify malignant melanoma, nevus, and seborrheic keratosis using the ISIC 2017 dataset. The average AUC was 84.8%, and the average accuracy was 83.8%. Mandache et al. [63] used a CNN to distinguish between BCC and non-BCC lesions on 40 FF-OCT images. Their system, consisting of 10 layers for feature extraction, achieved an accuracy of 95.93%, a sensitivity of 95.2%, and a specificity of 96.54%. Hasan et al. [64] used a CNN on the ISIC database to classify benign and malignant lesions. They employed the ABCD symptomatic checklist for feature extraction, achieving an accuracy of 89.5%. In a study by Kanimozhi et al. [65], they aimed to classify melanoma using an artificial neural network (ANN) with a backpropagation algorithm. The dataset consisted of 31 dermoscopic images, and they used the ABCD parameters for feature extraction, achieving an accuracy of 96.9%. Another study [66] utilized a backpropagation neural network as a classifier to distinguish malignant from non-malignant lesions using 448 mixed-type images. They employed ROI and SRM for segmentation, achieving an accuracy of 70.4%.

Generative Adversarial Networks (GANs) are machine learning models consisting of a generator that creates synthetic data and a discriminator that evaluates its authenticity. For skin cancer detection, GANs can generate additional synthetic images to augment limited datasets, improving the performance of diagnostic models. They can also enhance image quality and assist in segmenting lesions, aiding more accurate diagnoses. A new data augmentation technique for skin lesions has been developed using a self-attention progressive GAN (PGAN) (43). Rashid et al. [67] proposed a GAN-based system for classifying AK, BCC, benign keratosis, dermatofibroma, melanoma, melanocytic nevus, and vascular lesions using the ISIC 2018 dataset. The system used a deconvolutional network and a CNN as generator and discriminator modules, achieving an accuracy of 86.1%. Bisla et al. [68] developed a deep convolutional GAN system for classifying melanoma, nevus, and seborrheic keratosis using the ISIC 2017, ISIC 2018, and PH2 datasets. They used decoupled deep convolutional GANs for data augmentation, achieving a ROC AUC of 91.5% and an accuracy of 86.1%. In a study by Vilimszky et al. [69], they developed a fully automatic ultrasound-based classification framework for skin cancer, comparing it with two semi-automated methods. They trained a support vector machine (SVM) on 310 lesions, achieving AUCs over 90% and ACCs over 85% for classifying nevi from cancerous lesions. This study uniquely assessed the impact of fully automated segmentation, finding it did not degrade performance by more than 5%.

Recently, some studies have been conducted to assess the contributions of chatbots in the interpretation of skin lesions. These chatbots utilize deep learning and convolutional neural networks trained on large datasets of dermoscopic images, enabling them to provide differential diagnoses for various skin conditions. They can identify lesions such as BCC, SCC, melanoma, and benign lesions. Chatbots can also generate reports that may support educational purposes for dermatologists. However, their performance can vary depending on the complexity of the dermoscopic language and specific clinical scenarios. In some cases, their diagnostic accuracy may be lower for certain types of lesions, such as inflammatory dermatoses, compared to more familiar conditions like BCC. The effectiveness of chatbots in dermatology depends on the quality of their training data and their ability to interpret detailed and nuanced dermoscopic descriptions [70].

The integration of AI into existing clinical workflows presents additional challenges. Healthcare professionals must adapt to new technologies, which requires substantial training and adjustment to their routines. The complexity of AI systems can also lead to a lack of transparency, making it difficult for clinicians to understand and trust AI-generated recommendations. This “black box” problem hinders the adoption of AI in clinical settings [71]. The anatomical complexity of head and neck tissues poses another unique challenge for AI applications. High-resolution imaging is crucial to capture the detailed features necessary for accurate diagnosis. Variability in tissue presentation, due to factors like patient age, underlying health conditions, and previous treatments, further complicates AI training. Additionally, the proximity of vital structures requires precision in diagnosis and treatment planning to avoid collateral damage [72].

The interpretability of AI decisions is another critical issue. Clinicians need to understand the rationale behind AI-generated conclusions to make informed decisions. Lack of interpretability can lead to mistrust and reluctance to rely on AI tools. Furthermore, the continuous evolution of AI technology means that systems require regular updates and retraining with new data to maintain their efficacy [73]. Training AI models involves several steps: data collection, preprocessing, feature extraction, and model training. High-quality, annotated datasets are essential for developing robust AI models. Diverse datasets that include various skin cancer types, stages, and presentations improve the generalizability of AI systems. Preprocessing steps, such as normalization and augmentation, enhance the dataset’s quality and variability, allowing the AI to learn more effectively [71]. Artifacts and poor tissue processing can significantly impact AI performance. Common artifacts, such as air bubbles, folding, and staining inconsistencies, can mislead AI models. Training AI to recognize and account for these artifacts involves incorporating them into the training dataset and developing algorithms that can distinguish between artifacts and true pathological features [74]. Standardizing biopsy procedures and histological processing can reduce variability and improve AI accuracy. Consistent use of fixatives, such as H&E, IHC, and immunofluorescence, ensures uniformity in tissue samples, facilitating better AI training and performance. Standardization helps in minimizing the discrepancies caused by different laboratory practices and enhances the reproducibility of AI diagnostic tools [75].

Immunohistochemistry plays a crucial role in enhancing AI training for skin cancer diagnostics by providing detailed molecular insights through monoclonal antibodies that target specific antigens in cancer cells. These antibodies are essential for identifying various skin tumors and include markers such as S-100, HMB-45, Melan-A, Cytokeratin 5/6, and Ber-EP4. S-100 is a protein marker highly sensitive for melanocytes and is widely used in the diagnosis of melanoma, although it is not entirely specific. HMB-45 targets gp100, a melanosome-associated protein, and is more specific for melanocytic lesions, making it critical for distinguishing melanoma [76]. Melan-A (MART-1) is another marker specific for melanocytes, often used alongside S-100 and HMB-45 to improve diagnostic accuracy for melanoma [77]. For SCC, Cytokeratin 5/6 is used, as it is expressed in the basal and suprabasal layers of the epidermis, aiding in differentiation from other skin tumors [78]. Ber-EP4 is commonly used for BCC as it stains epithelial cell adhesion molecules, helping to differentiate BCC from SCC [79]. Incorporating IHC data into AI training datasets allows AI systems to learn the specific features associated with different cancer types, thereby improving diagnostic accuracy. Standardizing IHC procedures ensures consistency in the data used for AI training, reducing variability and enhancing the reproducibility of AI models. By leveraging the detailed molecular information provided by IHC, AI systems can achieve higher diagnostic accuracy, aiding in early detection and improving patient outcomes. [80,81].

Despite the promising advancements, several challenges remain in the widespread adoption of AI in clinical settings. One significant challenge is the need for high-quality data to train AI models. Large datasets are essential for developing robust AI algorithms that accurately distinguish between benign and malignant lesions. Efforts are ongoing to collect and curate such data to enhance the reliability of AI-based diagnostic tools. Additionally, integrating AI into routine clinical workflows requires careful consideration of human–AI interaction. Clinicians’ acceptance and trust in AI recommendations are critical for successfully implementing these technologies. Studies have shown that while AI can complement human judgment, particularly in making complex diagnostic decisions, it is best used as an assistive tool rather than a replacement for expert clinicians.

## 4. Conclusions

AI has demonstrated significant potential in improving skin cancer diagnosis through enhanced accuracy and the integration of diverse diagnostic data. While challenges remain, ongoing research and development will likely address these issues, paving the way for AI to become an integral part of skin cancer diagnostic practice. The ultimate goal is to leverage AI to provide more accurate, efficient, and personalized care for patients with skin cancer.

## Figures and Tables

**Table 1:** Skin cancer diagnosis methods.

Diagnosis Method	Description	Advantages	Limitations
**Visual Examination**	Dermatologists examine skin visually for suspicious moles or lesions.	Non-invasive, quick, and accessible	Subjective potential for human error
**Dermatoscopy**	A handheld device (dermatoscope) magnifies and illuminates the skin.	Provides more detail than visual examination	Requires training, interpretation can still be subjective
**Biopsy**	Removal of a sample of skin tissue for laboratory analysis.	Definitive diagnosis	Invasive, may cause scarring, time-consuming
**Histopathology**	Microscopic examination of biopsied tissue by a pathologist.	Detailed analysis, can determine cancer type and stage	Requires biopsy, time-consuming
**Molecular Pathology**	Analysis of genetic and molecular markers in the skin tissue.	Can provide information on genetic mutations and prognosis	Requires specialized equipment, expensive
**High-Frequency Ultrasound**	Uses sound waves to create images of skin layers.	Non-invasive, real-time imaging	Limited penetration depth, operator-dependent
**Reflectance Confocal Microscopy**	Provides high-resolution images of the skin’s epidermis and upper dermis.	Non-invasive, can be used in vivo	Expensive, requires specialized training
**Optical Technique**	Uses light to detect skin abnormalities.	Non-invasive, real-time results	Limited depth penetration, affected by skin pigmentation
**Photodynamic-Based Technique**	Uses photosensitizing agents and light to detect and treat cancer.	Can target specific cancer cells, minimally invasive	Requires specialized agents, can cause skin sensitivity
**Thermal Imaging Technique**	Detects heat patterns and blood flow in tissues.	Non-invasive, can detect abnormal blood flow	Limited by resolution, affected by external temperature
**Spectroscopy**	Measures the interaction of light with tissue to identify abnormalities.	Non-invasive, can provide molecular information	Requires specialized equipment, interpretation complexity
**Multispectral Imaging Technique**	Uses multiple wavelengths of light to capture detailed images.	Provides comprehensive data, can identify different tissues	Requires complex analysis, expensive
**Computed Tomography (Ct)**	Uses X-rays to create detailed cross-sectional images of the body.	High-resolution images, useful for advanced cases	High radiation dose, expensive
**Magnetic Resonance Imaging (Mri)**	Uses magnetic fields and radio waves to create detailed images.	No radiation, excellent soft tissue contrast	Expensive, time-consuming, and requires patient cooperation
**Digital Pathology**	Digitization of pathology slides for AI-assisted analysis and sharing.	Enables remote consultations, enhances collaboration	Requires infrastructure for digitization and data storage
**AI-** **Based Image Analysis**	AI algorithms analyze images of skin lesions to identify malignancies.	High accuracy, can process large volumes of data quickly	Requires large datasets for training, potential biases
**Radiomics**	Extracts quantitative features from radiographic images for analysis.	Can identify patterns not visible to the naked eye	Requires advanced software and expertise

**Table 2:** AI models and their applications in skin cancer detection.

AI Model	Types of Skin Cancer Detected	Description
**CNN**	Melanoma, Basal Cell Carcinoma, Squamous Cell Carcinoma	CNNs are widely used for image recognition tasks. In skin cancer detection, they analyze dermoscopic images to identify malignant lesions.
**ResNet**	Melanoma, Basal Cell Carcinoma, Squamous Cell Carcinoma	ResNet is known for its deep layers and ability to overcome the vanishing gradient problem, making it effective for detailed image analysis in detecting skin cancer.
**Inception Network**	Melanoma, Basal Cell Carcinoma	Inception Networks (e.g., Inception-v3) utilize multiple convolutional filters at different scales, enhancing the model’s ability to detect various skin cancer types from images.
**Mobilenet**	Melanoma, Basal Cell Carcinoma	MobileNet is optimized for mobile and embedded vision applications, making it suitable for portable skin cancer detection tools.
**Densenet (Densely Connected Networks)**	Melanoma, Basal Cell Carcinoma, Squamous Cell Carcinoma	DenseNet connects each layer to every other layer in a feed-forward fashion, improving the flow of information and gradients throughout the network.
**Svm (Support Vector Machine)**	Melanoma, Basal Cell Carcinoma	An SVM is a supervised learning model used for classification. In skin cancer detection, it works by finding the hyperplane that best separates different types of skin lesions.
**Random Forest**	Melanoma, Basal Cell Carcinoma	Random Forest is an ensemble learning method that constructs multiple decision trees. It is used to improve the accuracy and robustness of skin cancer detection models.
**YOLO (** **You Only Look Once)**	Melanoma	YOLO is a real-time object detection system that processes images quickly, making it suitable for rapid skin cancer screening in clinical settings.

## Data Availability

Data sharing is not applicable.

## References

[1] Scarabello A., Muti P. (2013). Epidemiology and prevention of cutaneous tumors. Skin Cancer: A Practical Approach.

[2] Guy G.P., Thomas C.C., Thompson T., Watson M., Massetti G.M., Richardson L.C. (2015). Vital signs: Melanoma incidence and mortality trends and projections—United States, 1982–2030. MMWR Morb. Mortal Wkly. Rep..

[3] Ciuciulete A.-R., Stepan A.E., Andreiana B.C., Simionescu C.E. (2022). Non-melanoma skin cancer: Statistical associations between clinical parameters. Curr. Health Sci. J..

[4] Netscher D.T., Leong M., Orengo I., Yang D., Berg C., Krishnan B. (2011). Cutaneous malignancies: Melanoma and nonmelanoma types. Plast. Reconstr. Surg..

[5] Nakayama M., Tabuchi K., Nakamura Y., Hara A. (2011). Basal cell carcinoma of the head and neck. J. Ski. Cancer.

[6] Thieu K., Ruiz M.E., Owens D.M. (2013). Cells of origin and tumor-initiating cells for nonmelanoma skin cancers. Cancer Lett..

[7] Marks R. (1995). An overview of skin cancers. Cancer.

[8] Shashanka R., Smitha B. (2012). Head and neck melanoma. Int. Sch. Res. Not..

[9] Kwa R.E., Campana K., Moy R.L. (1992). Biology of cutaneous squamous cell carcinoma. J. Am. Acad. Dermatol..

[10] Ouyang Y.-H. (2010). Skin cancer of the head and neck. Semin. Plast. Surg..

[11] Reichrath J., Leiter U., Eigentler T., Garbe C. (2014). Epidemiology of skin cancer. Sunlight Vitam. D Ski. Cancer.

[12] Johnson T.M., Rowe D.E., Nelson B.R., Swanson N.A. (1992). Squamous cell carcinoma of the skin (excluding lip and oral mucosa). J. Am. Acad. Dermatol..

[13] Buzzell R.A. (1993). Effects of solar radiation on the skin. Otolaryngol. Clin. N. Am..

[14] Lázár P., Molnár E.T., Bende B., Vass G., Baltás E., Paczona R., Varga E., Piffkó J., Kemény L., Oláh J. (2023). Challenges in the complex management of neglected cutaneous melanomas in the head and neck area: A single center experience. J. Clin. Med..

[15] Apalla Z., Nashan D., Weller R.B., Castellsagué X. (2017). Skin cancer: Epidemiology, disease burden, pathophysiology, diagnosis, and therapeutic approaches. Dermatol. Ther..

[16] Shumrick K.A., Coldiron B. (1993). Genetic syndromes associated with skin cancer. Otolaryngol. Clin. N. Am..

[17] Kim R.Y., Ward B.B., Zide M.F. (2022). Head and Neck Skin Cancer. Peterson’s Principles of Oral and Maxillofacial Surgery.

[18] Alam M., Ratner D. (2001). Cutaneous squamous-cell carcinoma. N. Engl. J. Med..

[19] Padgett J.K., Hendrix J.D. (2001). Cutaneous malignancies and their management. Otolaryngol. Clin. N. Am..

[20] Kocaaslan F.N.D., Alakus A.C., Sacak B., Celebiler O. (2019). Evaluation of residual tumors and recurrence rates of malignant melanoma and non-melanoma skin cancer of head and neck region. Marmara Med. J..

[21] Langley R., Barnhill R., Mihm M., Fitzpatrick T., Sober A. (2003). Neoplasms: Cutaneous melanoma. Fitzpatrick’s Dermatol. Gen. Med..

[22] Pampena R., Kyrgidis A., Lallas A., Moscarella E., Argenziano G., Longo C. (2017). A meta-analysis of nevus-associated melanoma: Prevalence and practical implications. J. Am. Acad. Dermatol..

[23] Pralong P., Bathelier E., Dalle S., Poulalhon N., Debarbieux S., Thomas L. (2012). Dermoscopy of lentigo maligna melanoma: Report of 125 cases. Br. J. Dermatol..

[24] Dinnes J., di Ruffano L.F., Takwoingi Y., Cheung S.T., Nathan P., Matin R.N., Chuchu N., Chan S.A., Durack A., Bayliss S.E. (2019). Ultrasound, CT, MRI, or PET-CT for staging and re-staging of adults with cutaneous melanoma. Cochrane Database Syst. Rev..

[25] Gandhi S.A., Kampp J. (2015). Skin cancer epidemiology, detection, and management. Med. Clin..

[26] Wolner Z.J., Yélamos O., Liopyris K., Rogers T., Marchetti M.A., Marghoob A.A. (2017). Enhancing skin cancer diagnosis with dermoscopy. Dermatol. Clin..

[27] Kraft S., Granter S.R. (2014). Molecular pathology of skin neoplasms of the head and neck. Arch. Pathol. Lab. Med..

[28] Tímár J., Ladányi A. (2022). Molecular pathology of skin melanoma: Epidemiology, differential diagnostics, prognosis and therapy prediction. Int. J. Mol. Sci..

[29] Bhatt K.D., Tambe S.A., Jerajani H.R., Dhurat R.S. (2017). Utility of high-frequency ultrasonography in the diagnosis of benign and malignant skin tumors. Indian J. Dermatol. Venereol. Leprol..

[30] Tamas T., Dinu C., Lenghel M., Băciuț G., Bran S., Stoia S., Băciuț M. (2021). The role of ultrasonography in head and neck Non-Melanoma Skin Cancer approach: An update with a review of the literature. Med. Ultrason..

[31] Myslicka M., Kawala-Sterniuk A., Bryniarska A., Sudol A., Podpora M., Gasz R., Martinek R., Kahankova Vilimkova R., Vilimek D., Pelc M. (2024). Review of the application of the most current sophisticated image processing methods for the skin cancer diagnostics purposes. Arch. Dermatol. Res..

[32] Catalano O., Roldán F.A., Varelli C., Bard R., Corvino A., Wortsman X. (2019). Skin cancer: Findings and role of high-resolution ultrasound. J. Ultrasound.

[33] Wortsman X. (2013). Sonography of facial cutaneous basal cell carcinoma. J. Ultrasound. Med..

[34] Bobadilla F., Wortsman X., Munoz C., Segovia L., Espinoza M., Jemec G.B. (2008). Pre-surgical high resolution ultrasound of facial basal cell carcinoma: Correlation with histology. Cancer Imaging.

[35] MacFarlane D., Shah K., Wysong A., Wortsman X., Humphreys T.R. (2017). The role of imaging in the management of patients with nonmelanoma skin cancer: Diagnostic modalities and applications. J. Am. Acad. Dermatol..

[36] Catalano O., Setola S.V., Vallone P., Raso M.M., D’Errico A.G. (2010). Sonography for locoregional staging and follow-up of cutaneous melanoma: How we do it. J. Ultrasound Med..

[37] Smith L., MacNeil S. (2011). State of the art in non-invasive imaging of cutaneous melanoma. Ski. Res. Technol..

[38] Lanka B., Turner M., Orton C., Carrington B.M. (2005). Cross-sectional imaging in non-melanoma skin cancer of the head and neck. Clin. Radiol..

[39] Rajabi-Estarabadi A., Bittar J.M., Zheng C., Nascimento V., Camacho I., Feun L.G., Nasiriavanaki M., Kunz M., Nouri K. (2019). Optical coherence tomography imaging of melanoma skin cancer. Lasers Med. Sci..

[40] Senan E.M., Jadhav M.E. (2019). Classification of dermoscopy images for early detection of skin cancer—A review. Int. J. Comput. Appl..

[41] Godoy S.E., Hayat M.M., Ramirez D.A., Myers S.A., Padilla R.S., Krishna S. (2017). Detection theory for accurate and non-invasive skin cancer diagnosis using dynamic thermal imaging. Biomed. Opt. Express.

[42] Haroon A., Shafi S., Rao B.K. (2017). Using reflectance confocal microscopy in skin cancer diagnosis. Dermatol. Clin..

[43] Onega T., Barnhill R.L., Piepkorn M.W., Longton G.M., Elder D.E., Weinstock M.A., Knezevich S.R., Reisch L.M., Carney P.A., Nelson H.D. (2018). Accuracy of digital pathologic analysis vs traditional microscopy in the interpretation of melanocytic lesions. JAMA Dermatol..

[44] Goyal M., Knackstedt T., Yan S., Hassanpour S. (2020). Artificial intelligence-based image classification methods for diagnosis of skin cancer: Challenges and opportunities. Comput. Biol. Med..

[45] Gillies R.J., Schabath M.B. (2020). Radiomics improves cancer screening and early detection. Cancer Epidemiol. Biomark. Prev..

[46] Attallah O., Sharkas M. (2021). Intelligent dermatologist tool for classifying multiple skin cancer subtypes by incorporating manifold radiomics features categories. Contrast Media Mol. Imaging.

[47] Ferlay J., Colombet M., Soerjomataram I., Parkin D.M., Piñeros M., Znaor A., Bray F. (2021). Cancer statistics for the year 2020: An overview. Int. J. Cancer.

[48] Leiter U., Keim U., Garbe C. (2020). Epidemiology of skin cancer: Update 2019. Sunlight Vitam. D Ski. Cancer.

[49] Housman T.S., Feldman S.R., Williford P.M., Fleischer Jr A.B., Goldman N.D., Acostamadiedo J.M., Chen G.J. (2003). Skin cancer is among the most costly of all cancers to treat for the Medicare population. J. Am. Acad. Dermatol..

[50] Rajpurkar P., Chen E., Banerjee O., Topol E.J. (2022). AI in health and medicine. Nat. Med..

[51] Zhang P., Kamel Boulos M.N. (2023). Generative AI in medicine and healthcare: Promises, opportunities and challenges. Future Internet.

[52] Takiddin A., Schneider J., Yang Y., Abd-Alrazaq A., Househ M. (2021). Artificial intelligence for skin cancer detection: Scoping review. J. Med. Internet Res..

[53] Fu’adah Y.N., Pratiwi N.C., Pramudito M.A., Ibrahim N. Convolutional neural network (CNN) for automatic skin cancer classification system. Proceedings of the IOP Conference Series: Materials Science and Engineering.

[54] Luchini C., Pea A., Scarpa A. (2022). Artificial intelligence in oncology: Current applications and future perspectives. Br. J. Cancer.

[55] Panda S., Padhi S., Gupta V., Suri J.S., Saxena S. (2024). Application and constraints of AI in radiomics and radiogenomics (RnR) studies of neuro-oncology. Radiomics and Radiogenomics in Neuro-Oncology.

[56] Huang S., Yang J., Fong S., Zhao Q. (2020). Artificial intelligence in cancer diagnosis and prognosis: Opportunities and challenges. Cancer Lett..

[57] Ali A.A., Al-Marzouqi H. Melanoma detection using regular convolutional neural networks. Proceedings of the 2017 International Conference on Electrical and Computing Technologies and Applications (ICECTA).

[58] Nasr-Esfahani E., Samavi S., Karimi N., Soroushmehr S.M.R., Jafari M.H., Ward K., Najarian K. Melanoma detection by analysis of clinical images using convolutional neural network. Proceedings of the 2016 38th Annual International Conference of the IEEE Engineering in Medicine and Biology Society (EMBC).

[59] Dorj U.-O., Lee K.-K., Choi J.-Y., Lee M. (2018). The skin cancer classification using deep convolutional neural network. Multimed. Tools Appl..

[60] Esteva A., Kuprel B., Novoa R.A., Ko J., Swetter S.M., Blau H.M., Thrun S. (2017). Dermatologist-level classification of skin cancer with deep neural networks. Nature.

[61] Mendes D.B., da Silva N.C. (2018). Skin lesions classification using convolutional neural networks in clinical images. arXiv.

[62] Harangi B., Baran A., Hajdu A. Classification of skin lesions using an ensemble of deep neural networks. Proceedings of the 2018 40th Annual International Conference of the IEEE Engineering in Medicine and Biology Society (EMBC).

[63] Mandache D., Dalimier E., Durkin J., Boceara C., Olivo-Marin J.-C., Meas-Yedid V. Basal cell carcinoma detection in full field OCT images using convolutional neural networks. Proceedings of the 2018 IEEE 15th International Symposium on Biomedical Imaging (ISBI 2018).

[64] Hasan M., Barman S.D., Islam S., Reza A.W. Skin cancer detection using convolutional neural network. Proceedings of the Proceedings of the 2019 5th International Conference on Computing and Artificial Intelligence.

[65] Kanimozhi T., Murthi A. (2016). Computer aided melanoma skin cancer detection using artificial neural network classifier. Singaporean J. Sci. Res. (SJSR) J. Sel. Areas Microelectron. (JSAM).

[66] Mahmoud M.K.A., Al-Jumaily A., Takruri M. The automatic identification of melanoma by wavelet and curvelet analysis: Study based on neural network classification. Proceedings of the 2011 11th International Conference on Hybrid Intelligent Systems (HIS).

[67] Rashid H., Tanveer M.A., Khan H.A. Skin lesion classification using GAN based data augmentation. Proceedings of the 2019 41St Annual International Conference of the IEEE Engineering in Medicine and Biology Society (EMBC).

[68] Bisla D., Choromanska A., Berman R.S., Stein J.A., Polsky D. Towards automated melanoma detection with deep learning: Data purification and augmentation. Proceedings of the IEEE/CVF Conference on Computer Vision and Pattern Recognition Workshops.

[69] Marosán-Vilimszky P., Szalai K., Horváth A., Csabai D., Füzesi K., Csány G., Gyöngy M. (2021). Automated skin lesion classification on ultrasound images. Diagnostics.

[70] Karampinis E., Toli O., Georgopoulou K.-E., Kampra E., Spyridonidou C., Roussaki Schulze A.-V., Zafiriou E. (2024). Can Artificial Intelligence “Hold” a Dermoscope?—The Evaluation of an Artificial Intelligence Chatbot to Translate the Dermoscopic Language. Diagnostics.

[71] Shortliffe E.H., Sepúlveda M.J. (2018). Clinical decision support in the era of artificial intelligence. JAMA.

[72] Mahmood H., Shaban M., Indave B., Santos-Silva A., Rajpoot N., Khurram S. (2020). Use of artificial intelligence in diagnosis of head and neck precancerous and cancerous lesions: A systematic review. Oral Oncol..

[73] Kelly C.J., Karthikesalingam A., Suleyman M., Corrado G., King D. (2019). Key challenges for delivering clinical impact with artificial intelligence. BMC Med..

[74] Bera K., Schalper K.A., Rimm D.L., Velcheti V., Madabhushi A. (2019). Artificial intelligence in digital pathology—New tools for diagnosis and precision oncology. Nat. Rev. Clin. Oncol..

[75] Saco A., Ramírez J., Rakislova N., Mira A., Ordi J. (2016). Validation of whole-slide imaging for histolopathogical diagnosis: Current state. Pathobiology.

[76] En el Diagnóstico M.I. (2021). Immunohistochemical markers in the differential diagnosis of melanoma and nevus in humans. Int. J. Morphol..

[77] Dass S.E., Huizenga T., Farshchian M., Mehregan D.R. (2021). Comparison of SOX-10, HMB-45, and melan-A in benign melanocytic lesions. Clin. Cosmet. Investig. Dermatol..

[78] Cives M., Mannavola F., Lospalluti L., Sergi M.C., Cazzato G., Filoni E., Cavallo F., Giudice G., Stucci L.S., Porta C. (2020). Non-melanoma skin cancers: Biological and clinical features. Int. J. Mol. Sci..

[79] Sunjaya A.P., Sunjaya A.F., Tan S.T. (2017). The use of BEREP4 immunohistochemistry staining for detection of basal cell carcinoma. J. Ski. Cancer.

[80] Dinehart M.S., Dinehart S.M., Sukpraprut-Braaten S., High W.A. (2020). Immunohistochemistry utilization in the diagnosis of melanoma. J. Cutan. Pathol..

[81] Van Herck Y., Antoranz A., Andhari M.D., Milli G., Bechter O., De Smet F., Bosisio F.M. (2021). Multiplexed immunohistochemistry and digital pathology as the foundation for next-generation pathology in melanoma: Methodological comparison and future clinical applications. Front. Oncol..

